# Intraspecific eye color variability in birds and mammals: a recent evolutionary event exclusive to humans and domestic animals

**DOI:** 10.1186/s12983-017-0243-8

**Published:** 2017-12-04

**Authors:** Juan J. Negro, M. Carmen Blázquez, Ismael Galván

**Affiliations:** 1 Estación Biológica de Doñana (CSIC), Avda. Americo Vespucio 26, 41092 Sevilla, Spain; 2 Centro de Investigaciones Biológicas del Noroeste (CIBNOR), 23096 La Paz, B.C.S Mexico

**Keywords:** Eye coloration, Iris, Domestication, Sexual dichromatism, OCA2 gene

## Abstract

**Background:**

Human populations and breeds of domestic animals are composed of individuals with a multiplicity of eye (= iris) colorations. Some wild birds and mammals may have intraspecific eye color variability, but this variation seems to be due to the developmental stage of the individual, its breeding status, and/or sexual dimorphism. In other words, eye colour tends to be a species-specific trait in wild animals, and the exceptions are species in which individuals of the same age group or gender all develop the same eye colour. Domestic animals, by definition, include bird and mammal species artificially selected by humans in the last few thousand years. Humans themselves may have acquired a diverse palette of eye colors, likewise in recent evolutionary time, in the Mesolithic or in the Upper Paleolithic.

**Presentation of the hypothesis:**

We posit two previously unrecognized hypotheses regarding eye color variation: 1) eye coloration in wild animals of every species tends to be a fixed trait. 2) Humans and domestic animal populations, on the contrary, have eyes of multiple colors. Sexual selection has been invoked for eye color variation in humans, but this selection mode does not easily apply in domestic animals, where matings are controlled by the human breeder.

**Testing the hypothesis:**

Eye coloration is polygenic in humans. We wish to investigate the genetics of eye color in other animals, as well as the ecological correlates.

**Implications of the hypothesis:**

Investigating the origin and function of eye colors will shed light on the reason why some species may have either light-colored irises (e.g., white, yellow or light blue) or dark ones (dark red, brown or black). The causes behind the vast array of eye colors across taxa have never been thoroughly investigated, but it may well be that all Darwinian selection processes are at work: sexual selection in humans, artificial selection for domestic animals, and natural selection (mainly) for wild animals.

**Electronic supplementary material:**

The online version of this article (10.1186/s12983-017-0243-8) contains supplementary material, which is available to authorized users.

## Background

Animal coloration has fascinated evolutionary ecologists ever since Darwin and Wallace debated about the contribution of natural and sexual selection to the evolution of ornamental characters. Considering vertebrates as a whole, eye color, and more specifically the color of the iris, is indeed a variable and conspicuous trait [1], encompassing practically every color of the rainbow [2], being in some species bright yellow or crimson red and in other species very dark and almost indistinguishable from the central pupil, which is always black. It seems, therefore, that some eye colors may facilitate advertising whereas other colors are less visible on the face or head, and may serve to conceal the eye [3]. Eye coloration may also be related to visual needs, as the pigments involved capture different light wavelengths [4]. None-the-less, the origin and functions of eye colors are still poorly understood [5].

## Presentation of the hypothesis

**Fig. 1:**
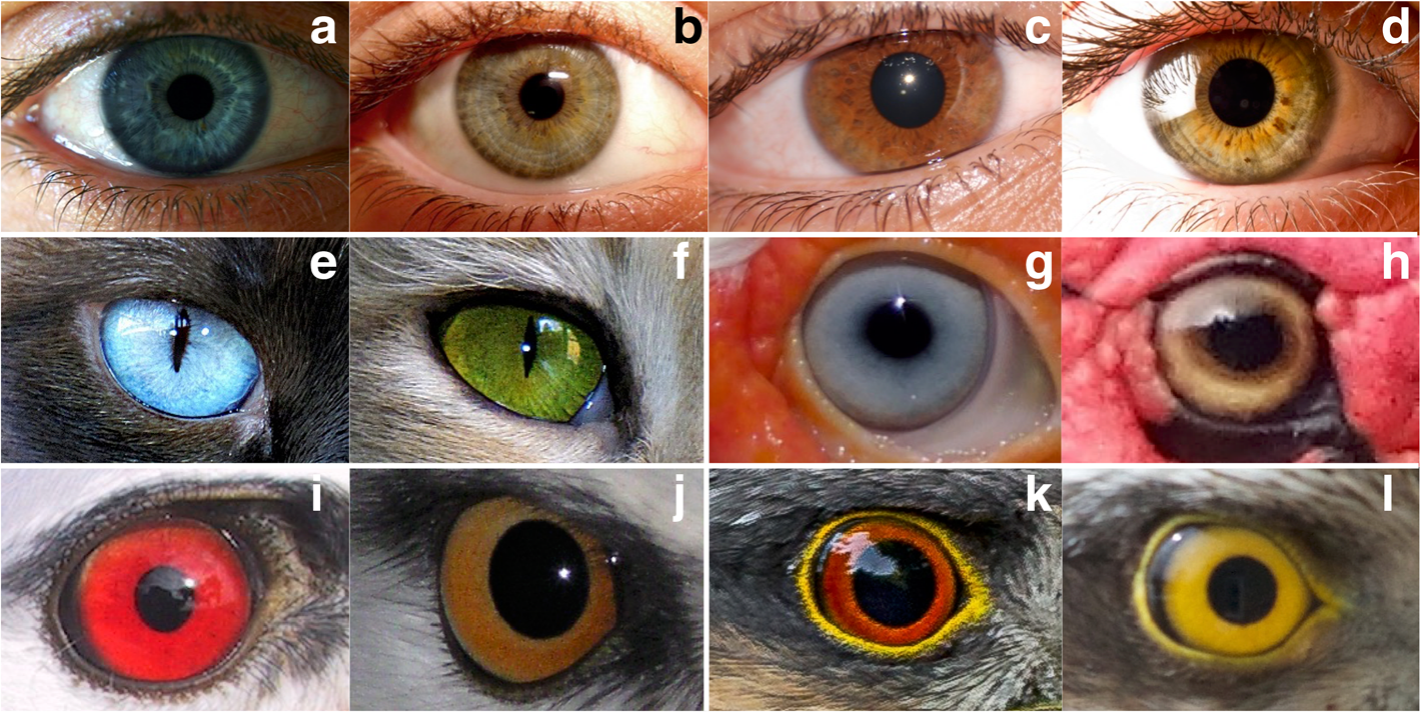
Iris color varies continuously in humans from very light blue to dark brown (upper line, **a**, **b**, **c**, **d**). Intrapopulational eye color variation is also characteristic of domestic animals (middle line): two adult cats *Felis catus* (**e**, **f**) and two adult domestic Muscovy ducks *Cairina moschata domestica* (**g**, **h**). In wild animals, however, iris color tends to be a fixed trait, with few observed variations due to maturation with age or sexual dichromatism. The bottom line shows color variation in two birds of prey. First a case of variation related to age in Black-winged kites *Elanus caeruleus* (**i**: adult; **j**: juvenile). Second, variation related to sex: Sparrowhawk *Accipiter nisus* (**k**: adult male; **l**: adult female). **a**: CC BY-SA 2.0 (https://creativecommons.org/licenses/by-sa/2.0/, Luisangel https://flic.kr/p/4vHKkh). **b**: CC BY 2.0 (https://creativecommons.org/licenses/by/2.0/, Gorgeous Eyes https://flic.kr/p/7vXh7G). **c**: CC BY 2.0 (https://creativecommons.org/licenses/by/2.0/, Jean-Simon Asselin https://flic.kr/p/2p4pFU). **d**: CC BY-SA 2.0 (https://creativecommons.org/licenses/by-sa/2.0/, Emilio Küffer https://flic.kr/p/agQvbv). **e**: CC BY 2.0 (https://creativecommons.org/licenses/by/2.0/, Trish Hamme https://flic.kr/p/dzfp8N). **f**: CC BY 2.0 (https://creativecommons.org/licenses/by/2.0/, Trish Hamme https://flic.kr/p/e8v8ro). **k**: CC BY 2.0 (https://creativecommons.org/licenses/by/2.0/, Andy Morffew https://flic.kr/p/KjN82N). **l**: CC BY 2.0 (https://creativecommons.org/licenses/by/2.0/, sighmanb https://flic.kr/p/ecc15G

We wish to report the fundamental observation that iris color variability is mainly interspecific and that, at the intraspecific level, animals in the avian and mammalian classes tend to have invariant species-specific coloration. This overlooked rule is broken by humans and domestic animals (in both the avian and mammalian classes), which display eye color variability at the species level (Fig. 1). Our working hypotheses are therefore two: 1) eye coloration in wild animals tends to be a fixed trait within species, the exceptions being eye color change during maturation, or associated with sexual dichromatism or breeding status. 2) Humans and domestic animals, on the contrary, have eyes of multiple colors at the species level. We will contend that this variation in humans and our domestic animals is recent in evolutionary time. This is evident with domestic animals whose ancestors underwent artificial selection in the Mesolithic or the Neolithic less than 10,000 years ago. But it may also be true for humans, as light colored eyes first appeared sometime before 8000 years, perhaps in northern Europe.

The genetic variant for blue eyes has been retrieved from DNA in hunter-gatherer remains at the Motala site in Sweden and Loschbour in Luxembourg, dated to 8000 years BP [6–8], but also in Mesolithic Europeans who lived in the Iberian Peninsula 7000 years ago [9]. All this evidence based on DNA suggests that multi-colored eyes emerged in *Homo sapiens* after their westward expansion in Europe [10], where they replaced the Neanderthals about 40,000 years ago [11]. In the case of domestic animals, light-colored eye variants became fixated necessarily in the Neolithic, or perhaps sometime before in the dog (*Canis lupus familiaris*), the first ever domesticated animal [12]. We know for certain that there is a difference in eye color in those species where the wild ancestors have persisted and show invariant eye color, as with cats and pigs. We must admit, however, that in those species for which the ancestor is now extinct, including cattle and horses, we can chart the diversification of eye color over time only by retrieving ancient DNA from remains.

## Testing the hypothesis

### Environmental correlates of eye coloration

In the scant literature on eye coloration in vertebrates, interspecific variation has been attributed not only to selection by the natural environment but also to predation pressure and sexual selection [5]. A study on more than 250 frog species in Madagascar found bright colored irises associated with tree frogs living in the forest canopy, whereas species with dark eyes would tend to be ground dwelling [13]. We know of no comparative studies for eye coloration of mammals, reptiles or fish. In birds, only two comparative studies have been published so far to our knowledge, both on passerines. One of them [14] reported an inexplicable higher proportion of bird species with bright irises in Southern Africa (25%) and Australia (35%) versus Canada (6%) and Europe (8%). The other study [5] reported that non-cavity nesting birds are under strong selection to evolve dark eyes, and that eye color was unrelated to parental care. At a general level, there is also the suggestion that stalking predators, whether mammals or birds, tend to have yellow or light-colored irises, whereas predators that run after their prey, and prey-species themselves, tend to be dark-eyed [2]. Despite the limited number of studies that explicitly look at variation in eye colour, they all suggest selection constrains variation within species.

Regarding intraspecific iris color variation, recognised factors for change in birds include maturation with age, changes during the mating season versus the non-breeding period, sexual dichromatism, and subspecies differentiation [15]. In the case of age-related changes in eye coloration, no information is available for wild mammals, although it is well known that baby humans change eye color in the first few months of life, and reaching the definitive eye color may take even longer. In wild birds, changes with age are relatively widespread [16], and typically involve species with brightly coloured irises as adults which, however, have dull-colored eyes as juveniles [17]. Sexually dimorphic eye coloration is rare in birds [5] and it is unknown in mammals, except for green eyes in humans, which are more frequent in women than in men [18–20].

We have conducted a survey on intraspecific color changes in birds due to either maturation with age, sexual dimorphism or breeding status (Additional files 1 and 2). The survey is, however, non-exhaustive, as it is based on published literature on single species, personal observations of species by the authors, and the verbal descriptions by M. Worthy [2] of 4918 species (about half of the species in Class Aves). This author based a majority of his descriptions on a literature review that he translated into a scale of eye darkness with five categories ranging from Yellow (0.00) to Black (1.00). For a great number of species, particularly those that are less studied and/or live in remote areas, no detailed information on eye colour of individuals of both sexes and/or different age classes is available. Eyes are not preserved in museum specimens and labels usually neglect to mention eye coloration at the time of capture.

**Fig. 2:**
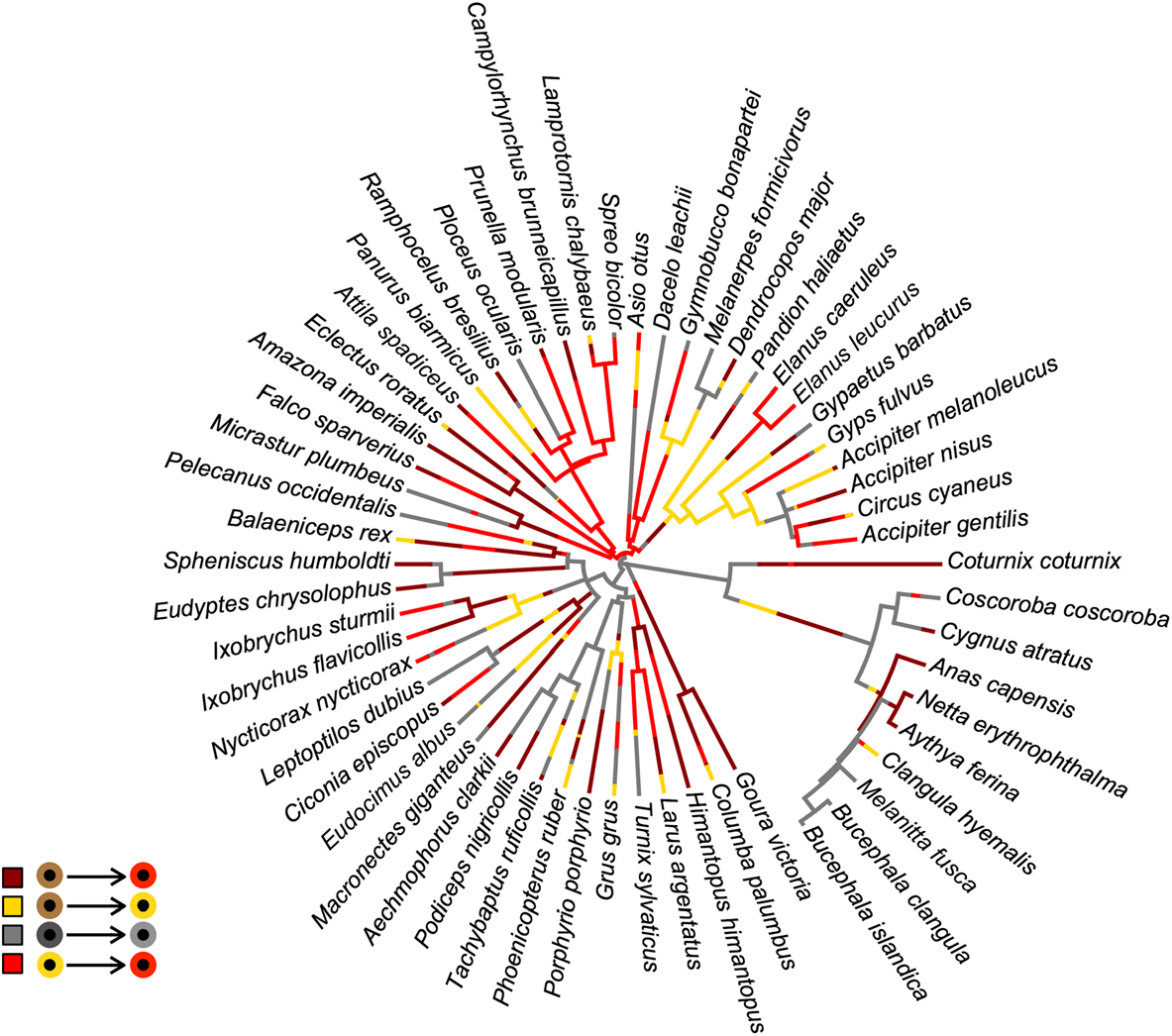
Phylogenetic relationships between 58 species of birds with four categories of age-related eye color changes (brown to red, brown to yellow, yellow to red and dark to light; depicted in the insert legend). The phylogenetic tree is the least-squares consensus tree calculated from the mean patristic distance matrix of a set of 1000 probable phylogenies from Jetz et al. 2012 (Nature 491: 444–448). The reconstruction of ancestral states was made by stochastic character mapping using an empirical Bayesian Monte Carlo Markov Chains (MCMC) approach (Huelsenbeck et al. 2003, Systematic Biology 52: 131–158) as implemented in *phytools* (Revell 2012, Methods in Ecology and Evolution 3: 217–223)

From our admittedly limited survey, we have determined that, if eye-color varies within species, a predictable change with age is the most usual cause of variation, in agreement with [16], and that this mode of variation is present across all extant bird lineages (see Fig. 2), including both non-passerines (at least 24 families) and passerines (at least 5 families). Color transitions go from a dark hue to a lighter/brighter one, from brown to yellow, from brown to red or from yellow to red, as advanced in [17]. The direction of these changes may reflect the sequential involvement of different pigments [4]: melanins would be more prevalent in brown-eyed juveniles, whereas other pigments that accumulate from the diet, such as carotenoids [21], would give their color to the adult eye. In most cases, eye coloration tends to be invariant in wild animals. Individuals of a particular species of the same sex and age display the same eye coloration. The only animals showing variability in eye coloration in the adult stage are domestic species. Further research is needed, however, to determine the actual extent of variation (i.e., hues and frequencies) in every domestic species.

### Genetic determinants of eye color variation

Human eye coloration is most diverse in Europe [22], but there is observable variability within brown eye colors elsewhere [23]. In fact, human eye color varies continuously from very light blues to very dark brown due to differences in melanin content in the iris [24, 25].

At the molecular level, much of the variation among light and dark eye coloration in humans is explained by a single mutation at the gene HERC2, which affects melanin production at the pigmentation gene known as OCA2, and it is responsible for an individual having brown eyes or non-brown eyes [10, 23]. None-the-less, recent investigations have identified that other genes have effects on iris pigmentation. For instance, the first genome-wide association study using quantitative eye color measurements from digital images instead of categorical eye color variation identified three new loci contributing to subtle differences in natural eye color, confirming that eye color is a polygenic trait [25]. No less than 50 SNPs (single nucleotide polimorphisms) are known to be associated with human eye color variation (https://www.snpedia.com/). The genetics behind eye color variation is still under intense investigation, and it is already apparent that eye color in humans is not an example of simple Mendelian inheritance as older textbooks state.

The molecular basis of eye coloration variation in domestic animals indicates that blue eyes and other light-colored phenotypes are often related to coat color mutations affecting melanin production. The pink eye dilution gene or p locus in mice, for instance, is responsible for light-colored eyes and it is an orthologue of the P gene in humans causing albinism [26]. In wild animals, genetic information is scant. One study [27] was aimed to determine whether blue eyes in wild lemurs had the same genetic basis as blue eyes in humans, and it found that the sequence of the homologous intron of the gene HERC2 is completely preserved in lemurs and other primates (except blue-eyed humans). Therefore blue eyes in humans and their distant primate relatives the lemurs is a phenotypic convergence. We urge further studies on how eye coloration is governed at the genetic level to understand selection pressures acting (or not acting) across species.

## Implications of the hypothesis

As stated above, only domestic animals have variable eye coloration. Light-colored individuals in otherwise dark colored species may arise as “de novo” mutations, such as a koala with blue eyes which recently made headlines as first in Australian history (http://www.dailymail.co.uk/news/article-508336/Meet-Frankie-rare-blue-eyed-baby-koala-named-Sinatra.html). Domesticated animals have diversified considerably from the original wild type in their coat or plumage coloration [28], and eye color has undergone a parallel process. Eye color variation in the domestic cat, for instance, is extraordinarily variable, with some eye color charts having more than 25 different eye colors (e.g., http://aminoapps.com/page/warriors/1546176/epidemiology-eye-pigmentation). Blue eyed individuals regularly occur in every domestic breed, from camels and goats to horses (Additional file 1: Figure S1).

Sexual dimorphism in eye coloration is comparatively rare in wild species [5], and in our survey (Additional files 1 and 2) we only report 25 species in 8 families, with a strong phylogenetic component, as there were 9 species of ducks and 4 birds of prey. Color changes associated with breeding status are even harder to find in wild vertebrates: we are aware of only 5 species of birds, including the brown pelican (*Pelecanus occidentalis*), which has light colored eyes during the breeding season, coincidental with the nuptial plumage, but returns to a drabber plumage and brown eyes in the non-breeding period (https://identify.whatbird.com/obj/62/identification/Brown_Pelican.aspx). In mammals, a unique situation involves the Arctic reindeer (*Rangifer tarandus*), which seasonally changes its eye color from golden to deep blue due to a modification of the tapetum lucidum in response to winter darkness [29]. These observations lead us to posit that iris color is not generally involved in sexual selection in wild animals and instead may be a trait under natural selection. To our knowledge, however, no study has identified all selection factors conducive to light or dark eye coloration (but see [5] for passerines).

For domestic animals we may exclude sexual selection as a driver for variation, as mating is arranged and directed by the human owner, and natural selection is also lessened due to protection from predators and artificial feeding. We thus hypothesize that eye-color diversity in dogs and other domestic animals is directly related to artificial selection. The preference of humans for rare phenotypes even ignores accompanying negative pleiotropic effects [28]. Blue-eyed dogs or alpacas, for instance, often have associated deafness, and would not easily survive in the wild.

In humans, it seems that the light-colored variants arose recently when our species spread into Europe from Africa [30]. Given that there is not a clear advantage in terms of vision capabilities to having blue eyes [31], there is the possibility that individuals with eye colors different from the presumably original dark eye phenotypes were actually preferred as mates, thus quickly spreading their alleles. This would be a case of sexual selection in action, known to favour color traits and color polymorphisms [22].

Eye coloration in wild animals is still an understudied puzzle. The molecular basis of human eye coloration is better understood than in any other animal taxon, but further research is needed to disentangle whether the apparent convergent evolution of multiple eye colorations in humans and our domesticated species is due to the same or different selection mechanisms.

## Additional files

Additional file 1: Figure S1. Eye color variation in domestic goats (Florida breed from Spain). Two blue-eyed individuals and two brown-eyed individuals from the same herd are shown in the picture. (JPEG 3789 kb)

Additional file 2: Table S1. Survey (non-exhaustive due to paucity of published information) of eye color changes in wild bird species. The species with demonstrated changes in eye coloration are less than 1% of all extant bird species, and in most instances, changes occur when young birds become adults (see, e.g., Nogueira DM, Alves MAS. Iris colour as an indicator of age feature in female Brazilian tanagers (Passeriformes: Emberizidae) confirmed by molecular sexing technique. Int. J. Trop. Biol. 2008; 56(4): 629–1633). (XLSX 23 kb)

## References

[1] Cott HB (1940). Adaptive coloration in animals.

[2] Worthy M. Animal eye colors: Authors Choice Press; 2000.

[3] Bortolotti GR, Hill GE, KJ MG (2006). Natural selection and coloration: protection, concealment, advertisement, or deception?. Bird coloration, vol. 2: mechanisms and measurements.

[4] Oliphant LW, Hudon J, Bagnara JT (1992). Pigment cell refugia in homeotherms. The unique evolutionary position of the iris. Pigment Cell Res.

[5] Davidson G, Thorton A, Clayton NS (2017). Evolution of iris color in relation to cavity nesting and parental care in passerine birds. Biol Lett.

[6] Mathieson I, Lazaridis I, Rohland N, Mallick S, Patterson N, Roodenberg SA, Harney E, Stewardson K, Fernandes D, Novak, Bermúdez de Castro JM, Carbonell E, Gerritsen F, Khokhlov A, Kuznetsov P, Lozano M, Meller H, Mochalov O, Moiseyev V, Rojo Guerra MA, Roodenberg J, Maria Vergès JM, Krause J, Cooper A, Alt KW, Brown D, Anthony D, Lalueza-Fox C, Haak W, Pinhasi R, Reich D. Genome-wide patterns of selection in 230 ancient Eurasians. Nature. 2015;528:499-503. 10.1038/nature16152 PMC4918750 26595274

[7] Anthrogenica. Surprising pale pigmentation in Mesolithic Motala HGs, Forum. 2015 http://www.anthrogenica.com/showthread.php?3975-Surprising-Pale-pigmentation-in-Mesolithic-Motala-HGs. Accessed 24 Nov 2017.

[8] Lazaridis I, Patterson N, Mittnik A, Renaud G, Mallick S (2013). Ancient human genomes suggest three ancestral populations for present-day Europeans. Nature.

[9] Olalde I, et al. Derived immune and ancestral pigmentation alleles in a 7,000-year-old Mesolithic European. Nature. 2014:225–8. 10.1038/nature12960 PMC4269527 24463515

[10] Eiberg H, Troelsen J, Nielsen M (2008). Blue eye color in humans may be caused by a perfectly associated founder mutation in a regulatory element located within the *HERC2* gene inhibiting *OCA2* expression. Hum Genet.

[11] Higham T (2014). The timing and spatiotemporal patterning of Neanderthal disappearance. Nature.

[12] Skoglund P (2015). Ancient wolf genome reveals an early divergence of domestic dog ancestors and admixture into high latitude breeds. Curr Biol.

[13] Amat F, Wollenberg KC, Vences M (2013). Correlates of eye color and pattern in mantelid frogs. Salamandra.

[14] Craig AJFK, Hulley PE (2004). Iris color in passerine birds: why be bright-eyed?. S Afr J Sci.

[15] Bortolotti GR, Smits J, Bird DM (2003). Iris color in American kestrels varies with age, sex, and exposure to PCBs. Physiol Biochem Zool.

[16] Wilson J, Hartley IR (2007). Changes in eye color of juvenile bearded tits *Panurus biarmicus* and its use in determining breeding productivity. Ibis.

[17] Nogueira DM, Alves MAS (2008). Iris colour as an indicator of age feature in female Brazilian tanagers (Passeriformes: Emberizidae) confirmed by molecular sexing technique. Int J Trop Biol.

[18] Frost P (2014). The puzzle of European hair, eye, and skin color. Adv Anthropol.

[19] Frost P, Kleisner K, Flegr J. Health status by gender, hair color, and eye color: Red-haired women are the most divergent. bioRxiv. 2017. 10.1371/journal.pone.0190238 PMC5746253 29284020

[20] Martinez-Cadenas C, Peña-Chilet M, Ibarrola-Villava M, Ribas G (2013). Gender is a major factor explaining discrepancies in eye colour prediction based on HERC2/OCA2 genotype and the IrisPlex model. Forensic Sci Int-Gen.

[21] Galván I, Garrido-Fernández J, Ríos J, Pérez-Gálvez A, Rodríguez-Herrera B, Negro JJ (2016). Tropical bat as mammalian model for skin carotenoid metabolism. PNAS.

[22] Frost P (2006). European hair and eye color. A case of frequency-dependent sexual selection?. Evol Hum Behav.

[23] Edwards M, Cha D, Krithika S, Johnson M, Cook G, Parra EJ (2016). Iris pigmentation as a quantitative trait: variation in populations of European, east Asian and south Asian ancestry and association with candidate gene polymorphisms. Pigment Cell Melanoma Res..

[24] Sturm RA, Larsson M (2009). Genetics of human iris color and patterns. Pigment Cell Melanoma Res.

[25] Liu F, Wollstein A, Hysi PG (2010). Digital quantification of human eye color highlights genetic Association of Three new Loci. PLoSGenet.

[26] Brilliant M (2001). The mouse p (pink-eyed dilution) and human P genes, Oculocutaneous albinism type 2 (OCA2), and Melanosomal pH. Pigment Cell Res.

[27] Bradley B, Pedersen A, Mundy NI (2009). Blue eyes in lemurs and humans: same phenotype, different genetic mechanism. Am J Phys Anthropol.

[28] Reissmann M, Ludwig A (2013). Pleiotropic effects of coat color-associated mutations in humans, mice and other mammals. Semin Cell Dev Biol.

[29] Stokkan KA (2013). Shifting mirrors: adaptive changes in retinal reflections to winter darkness un Arctic reindeer. Proc R Soc B.

[30] Templeton AR (2002). Out of Africa again and again. Nature.

[31] Donnelly MP (2012). A global view of the OCA2-HERC2 region and pigmentation. Hum Genet.

