# Supplementary figures and images for: Intraspecific eye color variability in birds and mammals: a recent evolutionary event exclusive to humans and domestic animals
Source: Front Zool. 2017 Dec 4;14:53. doi: 10.1186/s12983-017-0243-8 (PMC5716259; doi:10.1186/s12983-017-0243-8)

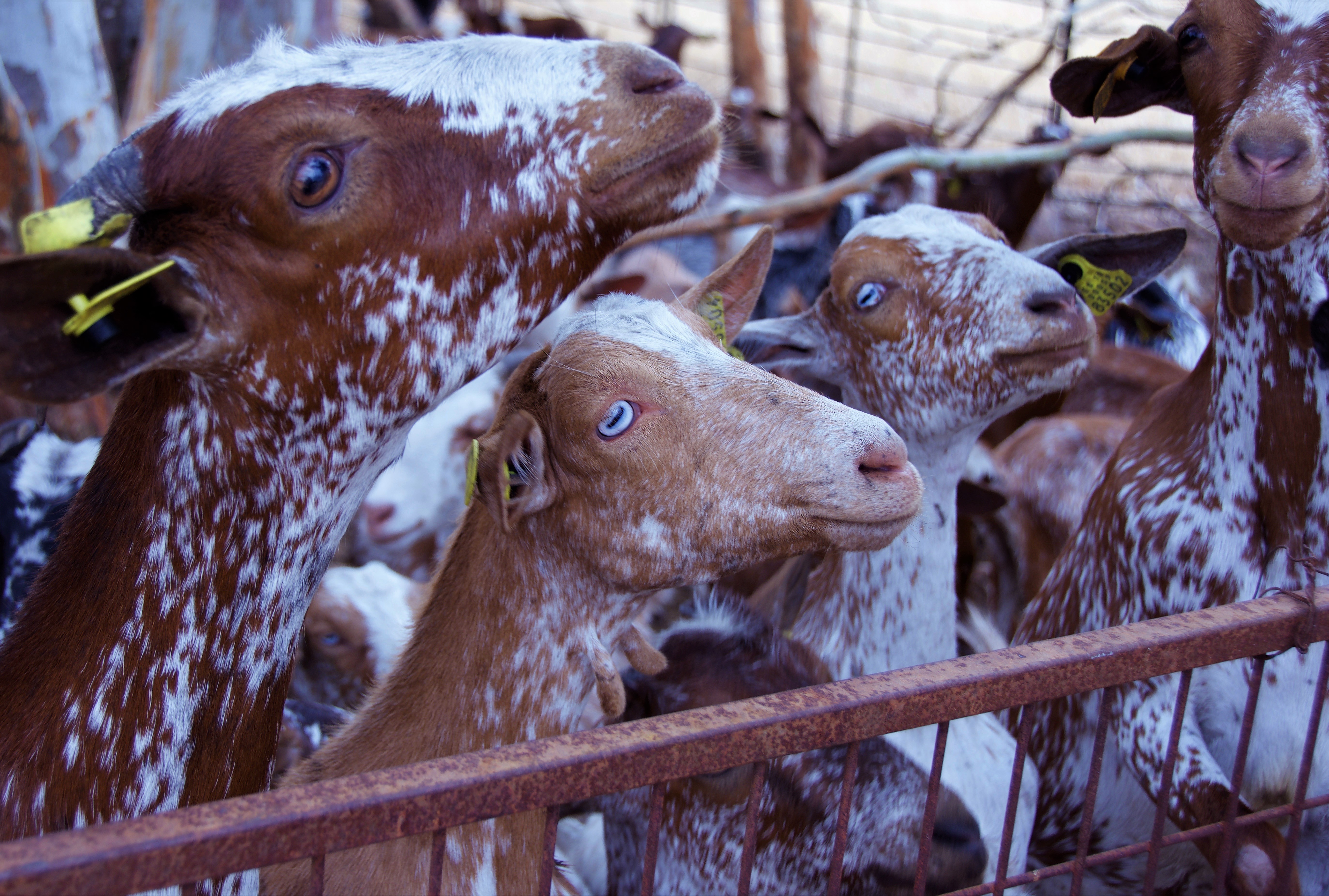

Supplement: Supplementary file 1 — Eye color variation in domestic goats (Florida breed from Spain). Two blue-eyed individuals and two brown-eyed individuals from the same herd are shown in the picture. (JPEG 3789 kb) [file 12983_2017_243_MOESM1_ESM.jpg]
